# Entrepreneurial Intentions of Teams: Sub-Dimensions of Machiavellianism Interact With Team Resilience

**DOI:** 10.3389/fpsyg.2019.02607

**Published:** 2019-11-21

**Authors:** Michaéla C. Schippers, Andreas Rauch, Frank D. Belschak, Willem Hulsink

**Affiliations:** ^1^Department of Technology and Operations Management, Rotterdam School of Management, Erasmus University Rotterdam, Rotterdam, Netherlands; ^2^University of Sydney Business School, Strategy, Innovation, and Entrepreneurship, University of Sydney, Sydney, NSW, Australia; ^3^Department of Leadership and Management, Amsterdam Business School, University of Amsterdam, Amsterdam, Netherlands; ^4^Department of Strategic Management and Entrepreneurship, Rotterdam School of Management, Erasmus University Rotterdam, Rotterdam, Netherlands

**Keywords:** Machiavellianism, team resilience, entrepreneurial intentions, team research, dark triad traits

## Abstract

Machiavellians are often seen as manipulative people who contribute negatively to teams and ventures. However, recent work has shown that Machiavellians can also cooperate and act in pro-social ways in a team context. Thus, some aspects of Machiavellianism might be conducive for teams and team members’ intentions to start a business venture. Most studies in this area have failed to (a) assess the effect of Machiavellianism at the team level, (b) take into account the dimensional nature of Machiavellianism, and (c) assess moderators of these effects. We propose that the combination of Machiavellianism and resilience in teams predict team entrepreneurial intentions (EI). Moreover, we propose that different team level dimensions of Machiavellianism (amoral manipulation, desire for status, desire for control, distrust of others) are differentially related to EI. More specifically, we expect at the team level that amoral manipulation and desire for status are positively related to changes in EI (as teams high on these dimensions feel that they can use unethical practices that give them an advantage in being successful), whereas desire for control and distrust of others should be negatively related to changes in EI (as entrepreneurial teams usually work in less structured situations and need to closely work together). Furthermore, all sub-dimensions of Machiavellianism should interact positively with team resilience as resilience acts as a buffer that protects teams from potential negative effects of Machiavellianism. In a multi-wave study among newly formed teams engaged in entrepreneurship projects, controlling for psychopathy and narcissism, we found partial support for our hypotheses. Results supported our expectations for the “amoral manipulation” and “desire for control” sub-dimensions, but not for the “desire for status” and the “distrust of others” sub-dimensions of Machiavellianism, with distrust of others showing unexpectedly opposite effects. This study contributes to the literature by looking at the dimensions of Machiavellianism at the level of entrepreneurial teams in conjunction with the more positive team characteristic, resilience. Our results indicate that the relationship between Machiavellianism and EI is more complex than previously hypothesized, as the sub-dimensions are sometimes positively and sometimes negatively related to entrepreneurial intentions and interact with team-level resilience.

## Introduction

Given that most new business ventures start out as a team (Lazar et al., 2019) and entrepreneurial education similarly emphasizes this team aspect (cf. Piperopoulos and Dimov, 2015), it seems imperative to assess factors that influence team-level entrepreneurial intentions. In particular there is a lack of research on individual differences in teams and how these affect team outcomes (Smith et al., 2018), and some individual differences may affect team outcomes in complex ways. For instance, people high on “dark personality traits” like Machiavellianism, can have a disruptive effect on team dynamics and outcomes as they tend to be manipulative and selfish (e.g., Wisse and Sleebos, 2016; Grijalva et al., 2019). At the same time, recent research has found that Machiavellians can also cooperate and act in a pro-social way when they believe it can be advantageous for them (Belschak et al., 2015). So more research is needed on how this personality variable affects teams under different circumstances.

Recently, the effects of the “dark triad personality traits” narcissism, Machiavellianism, and psychopathy have received increasing attention in the field of organizational psychology (e.g., Grijalva et al., 2015; Smith et al., 2018). Yet, the role of such dark traits in an entrepreneurial (team) context has not been subject to many studies so far and has just emerged as a new field (e.g., Miller, 2015; Klotz and Neubaum, 2016). Many new firms fail within a relatively short period of time. While some researchers such as Wasserman (2012) have referred to “people problems” in the formation of start-ups (i.e., differences in terms of goals, commitments, and incentives) and the pitfalls of founding a business with friends and family members as a potential explanation of their collapse, others have attributed such failures to an absence of positive personality traits in new venture teams (Klotz et al., 2014). Relatedly, Winslow and Solomon (1987, p. 206–207) identified entrepreneurs as “not necessarily mentally ill, but aberrant” and “mildly sociopathic.” A recent review on the upsides to dark and downsides to bright personality traits noted the nearly complete absence of studies at the team level incorporating dark personality (Smith et al., 2018). Indeed the few studies on entrepreneurship and dark traits have only discussed the role of such traits at the individual level (Hisrich et al., 2007; DeNisi, 2015). For instance, individual-level dark triad traits have been associated with the adoption of a “fast-life approach,” with a short-term orientation (Jonason et al., 2010), and with risky endeavors such as intending to start a business (Hmieleski and Lerner, 2016).

Of the three dark triad traits, Machiavellianism is the oldest construct and describes a person who is willing to use all possible means to achieve one’s ends, including unethical ones (Christie and Geis, 1970). Compared to its dark siblings, narcissism and psychopathy, Machiavellianism seems to be of particular relevance for a business and entrepreneurial context as Machiavellianism is the only personality trait that was not derived from a personality disorder in clinical psychology but rather was developed in an organizational and political context and generalized to social behavior (Fehr et al., 1992). This trait has been consistently linked to behaviors that affect team outcomes like counterproductive work behavior (O’Boyle et al., 2012), opportunistic behavior (Sakalaki et al., 2007), and unethical decision making with a focus on self-interest (Kish-Gephart et al., 2010). Nevertheless, Machiavellianism has been hardly applied to the realm of entrepreneurship, and some fundamental questions that we aim to address in our study still remain unanswered. How is team-level Machiavellianism related to team entrepreneurial intentions? Do the different sub-dimensions of Machiavellianism (amoral manipulation, desire for status, desire for control, and distrust of others; see Dahling et al., 2009) have similar effects on team outcomes, or do they produce differential effects? And how detrimental or useful is Machiavellianism (and its sub-dimensions) in combination with positive characteristics (specifically resilience) of entrepreneurial teams in predicting entrepreneurial intentions?

The intention to become an entrepreneur is likely to be affected by positive as well as negative personality characteristics. Entrepreneurial intentions are often seen in a positive light, and many predictors have been identified, such as proactive personality (Crant, 1996), Big Five personality traits (Zhao et al., 2010), and risk propensity (for meta-analyses see Frese et al., 2012). These results suggest that personality plays an important role in predicting entrepreneurial intentions. Recent research has noted that entrepreneurial intentions may also stem from less positive or idealistic motives, such as Machiavellianism. One study indeed supported the assumption that the dark triad – also referred to as the James Bond personality type (Jonason et al., 2009) – is related to entrepreneurial intentions and motives (Hmieleski and Lerner, 2016). Specifically, the authors found dark triad traits to be positively associated with unproductive entrepreneurial motives (e.g., maximizing profits, even at the expense of society, and/or employee wellbeing), and with productive entrepreneurial motives in students (e.g., generating value for society). Our research builds on this study and contributes to the literature in several ways.

First, we need additional studies to replicate and extend the results of Hmieleski and Lerner (2016). Moreover, these authors have focused on the individual level and thus research is needed into the positive and negative effects on entrepreneurial competencies and intentions at the team level (cf. LePine et al., 2011; Smith et al., 2018). Recently, Morris et al. (2013) have redefined the competencies needed by an entrepreneur; they have gone beyond the standard business functions that are vital for day-to-day company operations (e.g., selling and bookkeeping) and have put forward a new set of skills geared specifically to the particular requirements of the entrepreneurial context such as networking skills and resilience. Machiavellianism may reinforce some of these competencies as Machiavellians make extensive use of manipulation tactics like ingratiation, manipulation, and persuasion (Fehr et al., 1992). Also, studies on Machiavellianism have shown that Machiavellianism is related to charismatic leadership (Deluga, 2001) and has been linked to positive leader characteristics such as self-confidence and conviction (Wilson et al., 1996). However, Machiavellianism could also impede the development of other competencies such as risk mitigation and maintaining a long-term social relationship.

Second, a next step in this stream of research should be to look at Machiavellianism in entrepreneurial *teams*. Research on the downside of entrepreneurial personality has focused largely on firms started by a single entrepreneur. Most firms, however, are established by a founding team (Chowdhury, 2005; Lazar et al., 2019). In the words of Gartner et al. (1994, p. 6): “The ‘entrepreneur’ in entrepreneurship is more likely to be plural rather than singular. The locus of entrepreneurial activity often resides not in one person, but in many.” Studying team-level characteristics is all the more important with regard to Machiavellianism as people with pronounced Machiavellianism may have a very disruptive effect on team dynamics (e.g., Grijalva et al., 2019).

Finally, we explore the contingencies of the link between Machiavellianism and entrepreneurial intentions. Here, we expect there to be interactions at the team level between Machiavellianism and team-level resilience (Blatt, 2009). Resilience is the ability to bounce back from hardship, adversity, or failure (Luthans et al., 2006). Machiavellianism may lead to negative team dynamics, for example, by increasing conflict within the team and reducing the team’s ability to resolve issues (O’Neill and Allen, 2014). Resilience might help teams with a high level of dark traits to continue achieving their goals, despite difficult team dynamics, thus acting as a buffer against potential negative effects of Machiavellianism. If so, resilience may provide opportunities for a training intervention.

Thus, our research model assumes that the composition of Machiavellianism of teams affects their entrepreneurial intentions. However, the effect of traits on intentions is not direct but depends on moderation processes. Specifically, we expect that team resilience affects the strength of the relationship between team Machiavellianism and team entrepreneurial intentions. In the next section, we develop our hypotheses. Notably, there is not much literature on team-level dark traits, so we draw heavily on individual-level literature on dark traits in general and Machiavellianism in particular to justify our propositions.

## Theory and Hypotheses

### Machiavellianism and Entrepreneurship

The discussion of the dark side of entrepreneurship was initially stimulated by Kets de Vries with his clinical perspective of entrepreneurial intentions and behaviors (e.g., entrepreneurs’ need for control, distrust, and desire for applause; de Vries, 1996) and has re-emerged more recently in research on dark triad traits of entrepreneurs (Hmieleski and Lerner, 2016). Specifically, Machiavellianism might be important in the entrepreneurial process as it encourages agentic striving at the expense of, or with a disregard for, the welfare of others (Jones and Paulhus, 2009).

Recent research suggests that facets of traits may be important, especially if these facets or sub-dimensions are expected to differentially relate to outcome variables (Judge et al., 2013). In particular Machiavellianism has been conceptualized as a multidimensional construct (e.g., Christie and Geis, 1970; Dahling et al., 2009). Although the trait of Machiavellianism is associated with manipulative and amoral tactics and with a cynical, untrusting view of human nature (Dahling et al., 2009), the status and manipulation-related facets may have a different effect than the controlling and trust-related facets. Despite the hypothesis that Machiavellian individuals favor the maximization of personal gain and short-term profits, and entrepreneurship is associated with self-regulation and delayed gratification, Hmieleski and Lerner (2016) found Machiavellianism to be unrelated to the intention to start a business venture. The fact that they used a unidimensional scale of Machiavellianism may have concealed the possibility that its facets may have differential effects on entrepreneurial intentions. Indeed, some aspects of Machiavellianism might be relevant to entrepreneurship and even be positively associated with entrepreneurial intentions (Klotz and Neubaum, 2016). For instance, individuals high on manipulation tendencies might be overrepresented in entrepreneurial teams as individuals low on manipulation could reinforce each other in their conviction that entrepreneurs often behave unethically which might prevent them from being interested in entrepreneurial activities.

In this respect, Machiavellianism and its prime measure, the Mach-IV scale, were originally conceptualized as covering several distinct dimensions or content areas, but these were unfortunately not clearly identifiable empirically at that time because of psychometric problems with the scale (Dahling et al., 2009). More recently, however, Dahling et al. (2009) distinguished and validated four dimensions of this construct using a new measure that aims specifically to overcome the weaknesses of other measures of Machiavellianism. *Amoral manipulation* implies a readiness to disregard conventional standards of morality and to value one’s own actions at the expense of others. *Desire for status* is associated with an eagerness to accumulate external indicators of success. *Desire for control* is a need for dominance and to minimize the power of others. Finally, *distrust of others* refers to a negative and cynical outlook toward other people, implying distrust of other people’s actions and of the potential negative implications of those actions for the self. We argue that the different sub-dimensions of Machiavellianism might affect entrepreneurial intentions and behaviors in different ways and should therefore be investigated separately. More specifically, amoral manipulation and desire for status are likely to be positively related to entrepreneurial intentions, while desire for control and distrust of others are likely to be dysfunctional in terms of developing such intentions.

*Amoral manipulation* should be positively related to the intention to become an entrepreneur, albeit probably via more unproductive motives (Hmieleski and Lerner, 2016) such as sabotaging the efforts of others in order to get ahead. Teams scoring high on this dimension are likely to aim more easily for being an entrepreneur because they have an advantage over others in this field as the relatively unstructured entrepreneurial situation allows them to easily use unethical business practices like manipulation (which they are willing and able to use; Christie and Geis, 1970) without being easily discovered. As individuals high in amoral manipulation could reinforce each other in their conviction that it is okay (or even necessary) for entrepreneurs to behave unethically, this could influence team level EI. On the other hand, individuals low in amoral manipulation could reinforce each other in their conviction that entrepreneurs often behave unethically which might prevent them from being interested in entrepreneurial activities. *Desire for status* is an aspect of Machiavellianism that might be valuable in entrepreneurship, as it implies a focus on monetary rewards (Jonason and Webster, 2010). As a consequence, people with a strong desire for status might prefer an entrepreneurial career as this type of career might provide high status and financial success. In fact, Machiavellians can be relatively successful in their career, particularly when they work in unstructured and high-autonomy situations (Sparks, 1994; see Jones and Paulhus, 2009). In addition, there are several empirical studies indicating that Machiavellianism is related to positive work outcomes in unstructured situations where there is a high level of autonomy and little monitoring by supervisors (Ricks and Fraedrich, 1999; Bagozzi et al., 2013). Entrepreneurs work in fairly unstructured situations, and specifically a high desire for status in teams might therefore be associated with stronger team level intentions to become an entrepreneur. Also, teams comprised of individuals with a high level of desire for status could highlight this aspect to other team members and increase the salience of this aspect to other team members hence influencing team level EI positively.

*Desire for control* should be related to less strong intentions to become an entrepreneur. Although people who score high on this dimension of Machiavellianism like to have power over others, they also find it important to have control over the situation (Dahling et al., 2009). Usually, entrepreneurship is associated with a high level of uncertainty and a low level of control over a situation. Since domination is not easily achieved in situations that involve high uncertainty, a high level of desire for control in teams will be negatively related to team level entrepreneurial intentions. As noted above, team members can influence each other by highlighting this aspect of entrepreneurship. *Distrust of others* should be negatively related to entrepreneurial intentions because starting and running a business venture usually requires social capital and support (Rauch et al., 2016). Thus, entrepreneurs need to be able to build reliable networks. However, Machiavellianism carries considerable interpersonal risk, as distrust and manipulation can at times cause damage to social exchange relationships. We expect distrust of others to be negatively related to entrepreneurial intentions, since it is hard to do business with people if one is fundamentally distrustful of others. People who score high on this dimension will, when asked to think about becoming an entrepreneur or starting a venture, be less inclined to do so, and this aspect may be further highlighted and reinforced by other team members. Thus we hypothesize that:

Hypothesis 1: The Machiavellian dimensions of amoral manipulation and desire for status in teams are positively related to entrepreneurial intentions.

Hypothesis 2: The Machiavellian dimensions of desire for control and distrust of others in teams are negatively related to entrepreneurial intentions.

### Interactions Between Machiavellianism and Resilience

Prior research has shown that both the team context and traits are important in predicting team behavior and outcomes (e.g., Tett and Burnett, 2003; Tasa et al., 2011; for a review see LePine et al., 2011). The concept of resilience in particular has received considerable attention, as it helps to explain an individual’s ability to create positive outcomes, even in the face of great adversity (Luthar et al., 2000). Recent research has also stressed that resilience is important at the team level (Blatt, 2009; Alliger et al., 2015; Bowers et al., 2017). At this level, resilience is assumed to be a capacity that helps teams repair themselves or rebound after setbacks, and such teams are thus less likely to be damaged by threatening situations (West et al., 2009). An entrepreneurial team with a high level of Machiavellianism might itself create adverse conditions, in that more conflicts might arise within the team and team members might become less willing to work together. Such negative team dynamics might even add to the uncertainty inherent in entrepreneurial tasks (McMullen and Shepherd, 2006). These teams will therefore sometimes be less likely to develop entrepreneurial intentions. However, resilience might act as a buffer, minimizing the negative effects of adverse team situations. Resilience is thus a resource that protects the individual and team (cf. Backmann et al., 2019). Examples of such buffering effects are well documented in the stress literature (Cohen and Wills, 1985).

We therefore expect that resilience will moderate the relationship between Machiavellianism and entrepreneurial intentions. First, resilience can reinforce the positive relationship between Machiavellianism and entrepreneurial intentions. Here, it might help to overcome the potential negative side effects of aspects of the Machiavellian personality. For example, team resilience might reduce undesirable behaviors resulting from the unproductive aspects of team amoral manipulation, and the unmitigated striving for success that is driven by desire for status. In this respect, team resilience might allow teams to control and keep in check the counterproductive aspects of their Machiavellian traits, thus enabling them to derive maximum benefit from the positive aspects of Machiavellianism. Second, resilience can also help with regard to negative relationships between Machiavellianism and entrepreneurial intentions. While desire for control will generally be negatively related to entrepreneurial intentions (compare above), it might be less harmful for entrepreneurial intentions in teams that are high in resilience, as resilience helps them to deal with stressful situations and the uncertainty associated with entrepreneurship. However, teams with low resilience will be less equipped to deal with this uncertainty and thus their strong desire for control does not fit well with the uncertainty that comes with entrepreneurship. Similarly, distrust of others might be less harmful in teams that have the capacity to stay positive despite of negative team dynamics. Here, resilience might help teams to overcome their negative expectations of others and give others more easily the “benefit of the doubt.” Resilient teams are more likely to realize that they need to collaborate with others and that collaboration will help them ultimately in achieving their goals, that is, resilience might help teams to focus on the productive and goal-achievement aspects of collaboration rather than on their distrust of others. Moreover, resilient team members are likely to influence each other positively through a process of group emotional contagion, or the transfer of moods among people in a group. This effect has been shown to affect team processes and outcomes (Barsade, 2002).

Therefore, we hypothesize that:

Hypothesis 3: Team-level resilience will moderate the relationship between Machiavellianism and entrepreneurial intentions such that it strengthens the positive relationships between amoral manipulation and desire for status on the one hand and entrepreneurial intentions on the other hand.

Hypothesis 4: Team-level resilience will moderate the relationship between Machiavellianism and entrepreneurial intentions such that it reduces the negative relationships between desire for control and distrust of others on the one hand and entrepreneurial intentions on the other hand.

## Materials and Methods

### Procedure

The participants in this study were drawn from a cohort of second-year BSc Business Administration students taking the Orientation to Entrepreneurship course at a Dutch university. Using student samples for predicting entrepreneurial intentions is appropriate for this type of research and has also been reported in prior research (Fayolle, 2006; Krueger, 2009; Hmieleski and Lerner, 2016). Data collection took place using online surveys, which were sent out twice by two research assistants. The first round of measurements was in the beginning of the teaching period (T0: January), assessing the personality constructs (dimensions of Machiavellianism and resilience) together with the control variables, followed by the second round (T1: June) in which we examined teams’ entrepreneurial intentions. We contacted the students by e-mail and asked them to complete the online questionnaires. We debriefed students about the purpose of the research afterward by disseminating a short note detailing our findings and tentative conclusions. Students had the opportunity to opt out from this research by e-mail, which only one student did. We removed the student from the database.

The course consisted of two parts: the first theoretical part was made up of regular classes introducing the students to the entrepreneurship literature and was completed with a final closed-book exam. After completing this part of the course, the students formed self-selected teams of three to four students to carry out the entrepreneurship projects, which centered around a number of team-based assignments. Although team members were not actively involved in building a business, these project teams were in many ways quite similar to real-life entrepreneurial teams, which can be defined as two or more individuals who have a significant financial interest and participate actively in the formation and development of the venture (Cooney, 2005). Students kept the same teammates for all the tasks involved in all the various entrepreneurship projects throughout the course and hence had to engage in these entrepreneurship projects together as a team.

Although the research design of this study was unique in that it investigated team-level entrepreneurial intentions, the course set-up is similar to the one used by Piperopoulos and Dimov (2015), as well as to the one used by Allen (2001). In our course, like Piperopoulos and Dimov’s study, we also separated between a theoretical and a practical part. The first block of the course Orientation to Entrepreneurship (January–March) focused on introducing different types of entrepreneurs and their projects and businesses, and the specific context in which they emerge, survive, and grow. The students completed this first part of the course with an individual sit-down exam, relying upon a mix of multiple choice and open questions. The exam grade made up 50% of the course’s final grade.

In the second block (April–June), the course literature was applied in three different group assignments (entrepreneurship projects), which consisted of creating a short audio-visual profile of a start-up founder (*nascent entrepreneur*), the systematic analysis of a published (auto)biography of a well-known entrepreneur (*Titan entrepreneur*), and the creation and analysis of a case about a regionally well-known and experienced high-impact entrepreneur (*Local Hero*) on the basis of two face-to-face interviews (with the entrepreneur and with an important stakeholder of the entrepreneur’s company). The first team assignment included making an audiovisual profile of a startup entrepreneur (which counted for 10% of the final grade). The second team assignment was a poster presentation and report of the entrepreneurial biography read (which counted for another 10% of the final grade). The third assignment included a presentation and report of the interviews conducted with the local hero entrepreneur and his/her stakeholder in the company (15% of the final grade). The final deliverable was completing an integrated 6,000 words essay covering the within-case and cross-case analyses of the different entrepreneurs selected for this course (i.e., nascent, high-impact, and titan entrepreneurs). This final essay amounted for the final 15% of the final grade for this course. In total, the team assignments accounted for 50% of the final grade. The partial and overall grades were determined on an absolute basis, i.e., each team could earn a high grade if they did well, but were not rank-ordered. Student teams with an ambitious goal, for instance, picking a rich entrepreneurial biography or selecting remarkable business founders or regional business builders, or showing an innovative approach in making these audiovisual profiles or original questions asked during the interviews, were overall rewarded with higher than average grades.

In addition to identifying the main reasons why certain people engage in entrepreneurship and how entrepreneurs learn from their successes and failures, the focus of these group projects was on getting insights about what entrepreneurs do when confronted with critical events (e.g., the bankruptcy of a supplier, the death of an investor) and how they effectively overcome them. It was the expectation in this practical part of the course that entrepreneurial (auto)biographies and (video)interviews are of great significance to undergraduate students, and that they would learn and get inspired by these role models and subsequently reflect on their entrepreneurial intentions as a team (cf. Rauch and Hulsink, 2015). The team composition and team dynamics would then determine the final entrepreneurial intentions, as argued above.

### Sample

The data collection took place during the course spanning 6 months. When we left out all the recidivists (students that had failed the course the year before had to take parts of it again in order to pass the full course) and those that dropped out of the course before doing any (resit) exams and/or completing all the course’s team projects, the number of students enrolled for the course was 535. Nearly all the teams were four-person groups (only one was a three-person group and one was a two-person group). The response rate for the first survey was 65% (348 students) and for the second survey it was 34% (183 students). Of these respondents, 63% were male and the average age was 20.19 years (*SD* = 1.05). In total 470 students allocated in 134 teams were enrolled on the entrepreneurship projects of this course. We had matched data from both surveys of 87 teams. Mean comparisons revealed no significant differences between the original and the final sample on any of the variables measured in the study.

### Measures

The survey measured the independent variables used in this study – Machiavellianism and resilience and the control variables narcissism, psychopathy, gender, and age – at the beginning of the course (T0) and entrepreneurial intentions at both T0 and at the end of the course (T1).

#### Machiavellianism

Machiavellianism (T0) was measured using the sixteen-item version of the Machiavellian Personality Scale (Dahling et al., 2009). All subscales were based on a Likert scale ranging from 1 (totally disagree) to 5 (totally agree). The subscale *amoral manipulation* consisted of five items. An example item is: “I would cheat if there was a low chance of getting caught” (Cronbach’s alpha is 0.84). The subscale *desire for status* consisted of three items. An example item is: “Status is a good sign of success in life” (Cronbach’s alpha is 0.83). The subscale *desire for control* consisted of three items. An example item is: “I enjoy having control over other people” (Cronbach’s alpha is 0.75). The subscale *distrust of others* consisted of five items. An example item is: “People are only motivated by personal gain” (Cronbach’s alpha is 0.81).

#### Resilience

Resilience (T0) was measured using the ten-item version of the Connor–Davidson Resilience Scale (Campbell-Sills and Stein, 2007). Example items are: “I am able to adapt to change” and “I can stay focused under pressure” (1 = totally disagree, 5 = totally agree; Cronbach’s alpha is 0.87).

#### Entrepreneurial Intentions

Entrepreneurial intentions were measured at two different time points, at T0 and T1. The T1 measure was used as the outcome variable, while the T0 measure served as a control variable, so that in effect we measured the increase in entrepreneurial intentions. We used the six-item measure from Liñán and Chen (2009). Example items are: “My professional goal is to become an entrepreneur” and “I am determined to create a firm in the future” (1 = totally disagree, 5 = totally agree; Cronbach’s alpha is 0.94 for T0 and 0.95 for T1). Since our dependent variable entrepreneurial intentions is at the team level, we calculated the intraclass correlations (ICCs) for this variable. The ICC(1) in T0 was 0.104, and in T1 it increased to 0.114. In general, values higher than 0.10 are satisfactory for aggregating data to the team level (LeBreton and Senter, 2008). The substantial ICC at T0 might be due to the fact that participants could self-select their teams. The increase in ICC from T0 to T1 means that teams became slightly more homogeneous due to the group dynamics during their work on the entrepreneurship projects.

#### Control Variables

We controlled for a variety of characteristics in order to rule them out as alternative explanations of the variation in team-level entrepreneurial intentions. These were gender, age, and entrepreneurial intentions at T0. We controlled for gender as women have a lower proclivity to engage in entrepreneurship. Similarly, there are differences in terms of age and entrepreneurial activity (Parker, 2009). Moreover, controlling for entrepreneurial intentions at T0 allows us to converge toward a causal interpretation of relationships (Cohen and Cohen, 1975). We also controlled for psychopathy and narcissism at T0. Machiavellianism, narcissism, and psychopathy have been labeled as the dark triad of personality as they conceptually and empirically overlap to some extent (e.g., being selfish and malevolent in interpersonal dealings; see Jones and Paulhus, 2009). As a consequence, researchers on Machiavellianism recommend to include and control for narcissism and psychopathy in any research on Machiavellianism in order to avoid ambiguous results (e.g., Paulhus and Williams, 2002; see Jones and Paulhus, 2009). These traits were measured using the subscales of psychopathy and narcissism from the Dark Triad Dirty Dozen (Jonason and Webster, 2010). The subscale psychopathy consisted of four items. An example item is: “I tend to be unconcerned with the morality of my actions” (1 = totally disagree, 5 = totally agree; Cronbach’s alpha is 0.80). The subscale narcissism also consisted of four items. An example item is: “I tend to want others to admire me” (1 = totally disagree, 5 = totally agree; Cronbach’s alpha is 0.84).

Finally, for all personality variables in the study (dark triad traits and resilience), we also added the variability of the trait (i.e., the standard deviation) since this is good practice in most articles that use team composition with respect to personality (for a meta-analysis, see Peeters et al., 2006).

## Results

### Data Aggregation

Since the present study focused on a group-level dependent variable (i.e., team-level entrepreneurial intentions), aggregation to the group level is the most appropriate strategy for analyzing the data (Kashy and Kenny, 2000). In addition, a recent review by LePine et al. (2011) of research on personality in teams has indicated that the mean is superior to other measures of personality. We therefore used the mean (i.e., the average; see also Barrick et al., 1998) of the team members’ scores to represent resilience, entrepreneurial intentions, and the dark triad traits at the team level while controlling for the standard deviations of these variables.

### Descriptive Statistics

Descriptive statistics and correlations for the study can be found in Table 1 (team level: below diagonal; individual level: above diagonal). At the level of the teams, entrepreneurial intentions at T0 were positively and strongly related to entrepreneurial intentions at T1 (*r* = 0.57, *p* < 0.01). Positive relationships were also found between resilience (*r* = 0.38, *p* < 0.01), amoral manipulation (*r* = 0.21, *p* < 0.05), desire for status (r = 0.28, p < 0.01), and narcissism (*r* = 0.22, *p* < 0.05) on the one hand, and entrepreneurial intentions at T1 on the other hand.

**TABLE 1:** Means, standard deviations, correlations, and Cronbach’s alphas (bold values on the diagonal).

**Variable**	***M***	***SD***	**1**	**2**	**3**	**4**	**5**	**6**	**7**	**8**	**9**	**10**	**11**	**12**	**13**	**14**	**15**	**16**	**17**	**18**
*M*			20.17	1.37	3.85		2.31		3.58		3.26		2.54		2.35		3.13		3.07	3.01
*SD*			1.49	0.48	0.50		0.83		0.74		0.90		0.77		0.88		0.86		1.02	1.05
1. Age	20.19	1.05	–	−0.11^∗^	0.11^∗^		−0.11^∗^		0.01		0.02		0.02		–0.05		–0.02		0.08	0.09
2. Gender^a^	1.36	0.41	–0.06	–	–0.10		–0.32^∗∗^		–0.05		–0.24^∗∗^		–0.19^∗∗^		–0.38^∗∗^		–0.23^∗∗^		–0.23^∗∗^	–0.30^∗∗^
3. Resilience	3.84	0.38	0.18^∗^	–0.10	**0.87**		–0.05		0.12^∗^		0.06		–0.10		0.06		0.07		0.24^∗∗^	0.34^∗∗^
4. Resilience SD	0.42	0.27	0.17	–0.04	–0.06	–														
**Machiavellianism**																				
5. Amoral manipulation	2.37	0.62	–0.13	–0.36^∗∗^	–0.12	0.06	**0.84**		0.21^∗∗^		0.39^∗∗^		0.38^∗∗^		0.46^∗∗^		0.37^∗∗^		0.12^∗^	0.14
6. Amoral manipulation SD	0.70	0.41	0.01	–0.11	0.04	0.26^∗∗^	0.28^∗∗^	–												
7. Desire for control	3.59	0.42	0.00	–0.11	0.01	–0.02	0.18^∗^	–0.04	**0.75**		0.39^∗∗^		0.19^∗^		0.28^∗∗^		0.41^∗∗^		0.04	0.05
8. Desire for control SD	0.72	0.37	0.05	0.11	0.06	–0.07	−0.21^∗^	0.02	–0.39^∗∗^	–										
9. Desire for status	3.34	0.62	0.08	–0.30^∗∗^	0.08	0.17	0.46^∗∗^	0.07	0.39^∗∗^	–0.32^∗∗^	**0.83**		0.25^∗∗^		0.37^∗∗^		0.54^∗∗^		0.15^∗^	0.20^∗^
10. Desire for status SD	0.75	0.42	0.01	0.18	0.24^∗^	0.06	−0.21^∗^	0.03	0.08	0.21^∗^	–0.32^∗∗^	–								
11. Distrust of others	2.56	0.58	0.02	–0.24^∗∗^	–0.33^∗∗^	0.23^∗^	0.36^∗∗^	0.05	0.17	–0.11	0.26^∗∗^	–0.04	**0.81**		0.29^∗∗^		0.17^∗∗^		0.13^∗^	–0.01
12. Distrust of others SD	0.67	0.35	–0.10	0.11	0.15	–0.03	0.06	0.17	−0.20^∗^	0.30^∗∗^	–0.15	0.04	0.04	–						
13. Psychopathy	2.37	0.60	– 0.03	–0.47^∗∗^	0.09	0.17	0.58^∗∗^	0.00	0.26^∗∗^	–0.17	0.46^∗∗^	–0.11	0.37^∗∗^	–0.13	**0.80**		0.38^∗∗^		0.16^∗∗^	0.12
14. Psychopathy SD	0.79	0.42	0.04	–0.05	0.25^∗∗^	–0.02	–0.03	0.04	–0.04	0.08	0.01	0.08	–0.06	0.21^∗^	0.07	–				
15. Narcissism	3.17	0.53	0.00	–0.23^∗∗^	0.12	–0.02	0.34^∗∗^	–0.01	0.39^∗∗^	–0.31^∗∗^	0.59^∗∗^	–0.14	0.08	–0.15	0.37^∗∗^	–0.17	**0.84**		0.17^∗∗^	0.21^∗∗^
16. Narcissism SD	0.78	0.42	0.09	–0.02	0.24^∗^	0.08	–0.04	0.05	0.04	0.11	–0.10	0.29^∗∗^	0.06	0.01	–0.03	0.22^∗^	−0.20^∗^	–		
17. Entrepreneurial intentions T0	3.20	0.70	0.24^∗∗^	–0.26^∗∗^	0.27^∗∗^	0.06	0.17^∗^	0.05	0.17	–0.16	0.31^∗∗^	0.04	0.20^∗^	–0.17	0.17	–0.18	0.24^∗∗^	0.15	**0.93**	0.81
18. Entrepreneurial intentions T1	3.01	0.87	0.04	–0.33^∗∗^	0.38^∗∗^	–0.04	0.21^∗^	–0.01	–0.08	0.03	0.28^∗∗^	0.04	–0.04	–0.05	0.08	0.00	0.22^∗^	0.15	0.57^∗∗^	**0.94**

*^*a*^1 = male, 2 = female. ^∗^p < 0.05; ^∗∗^p < 0.01. Pairwise deletion of cases. Numbers above the diagonal are at the individual level; numbers below the diagonal are at the team level.*

### Hypotheses Testing

Prior to the hierarchical regression analyses testing our hypotheses, all continuous independent variables were standardized using Z-scores. Hypothesis 1 predicted that amoral manipulation and desire for status would be positively related to entrepreneurial intentions. Our predictions were partially corroborated: results showed that amoral manipulation was positively related to entrepreneurial intentions (β = 0.34, *p* < 0.05, see Table 2), but the link between desire for status and entrepreneurial intentions, although in the expected direction, failed to reach significance (β = 0.28, ns).

**TABLE 2:** Hierarchical regressions with the dependent variable entrepreneurial intentions T1 (*N* = 87 teams).

	**Model 1**			**Model 2**			**Model 3**		
**Variable**	**β**	***B***	***SE***	**β**	***B***	***SE***	**β**	***B***	***SE***
**Control variables**									
Age	–0.16	–0.11	0.13	–0.19	–0.13	0.12	–0.11	–0.08	0.11
Gender (dummy)^a^	−0.27^∗^	−0.26^∗^	0.11	–0.26^∗∗^	–0.25^∗∗^	0.10	–0.34^∗∗^	–0.34^∗∗^	0.08
Entrepreneurial Intentions T0	0.70^∗∗^	0.55^∗∗^	0.13	0.56^∗∗^	0.44^∗∗^	0.12	0.64^∗∗^	0.50^∗∗^	0.10
Psychopathy Mean	–0.22	–0.17	0.14	−0.38^∗^	−0.30^∗^	0.15	–0.58^∗∗^	–0.45^∗∗^	0.14
Psychopathy SD	0.02	0.03	0.09	–0.06	–0.07	0.08	0.01	0.01	0.07
Narcissism Mean	0.21	0.15	0.14	0.06	0.04	0.15	–0.18	–0.13	0.14
Narcissism SD	0.08	0.09	0.09	0.04	0.04	0.08	–0.09	–0.11	0.07
**Machiavellianism SD**									
Amoral Manipulation SD	–0.05	–0.05	0.08	–0.15	–0.17	0.08	–0.37^∗∗^	–0.42^∗∗^	0.09
Desire for status SD	0.02	0.03	0.08	0.11	0.13	0.08	0.37^∗∗^	0.42^∗∗^	0.09
Desire for control SD	0.12	0.14	0.08	0.10	0.12	0.08	0.22^∗∗^	0.25^∗∗^	0.07
Distrust of others SD	0.02	0.03	0.09	–0.04	–0.05	0.08	–0.04	–0.04	0.07
Resilience SD	–0.05	–0.06	0.08	0.00	0.00	0.08	0.14^∗^	0.17^∗^	0.07
**Main effects**									
**Machiavellianism**									
Amoral manipulation (AM)				0.34^∗^	0.29^∗^	0.15	0.65^∗∗^	0.54^∗∗^	0.15
Desire for status (DS)				0.28	0.22	0.15	0.39^∗∗^	0.30^∗∗^	0.14
Desire for control (DC)				−0.34^∗^	−0.22^∗^	0.15	−0.28^∗^	−0.19^∗^	0.13
Distrust of others (DO)				–0.12	–0.10	0.12	–0.05	–0.04	0.10
Resilience				0.31^∗^	0.27	0.13	–0.01	–0.01	0.13
**Two-way interaction**									
Resilience × AM							0.53^∗∗^	0.45^∗∗^	0.16
Resilience × DS							0.31	0.17	0.19
Resilience × DC							0.82^∗∗^	0.33^∗∗^	0.21
Resilience × DO							−0.21^∗^	−0.21^∗^	0.08
*R*^2^	0.44			0.58			0.72		
Δ*R*^2^	0.44			0.15			0.14		
Δ*F*	4.74^∗∗^			4.87^∗∗^			8.33^∗∗^		
*dfs*	(12, 74)			(5, 69)			(4, 65)		

*^*a*^1 = male, 2 = female. ^∗^p < 0.05, ^∗∗^p < 0.01.*

According to Hypothesis 2, both desire for control and distrust of others would be negatively related to entrepreneurial intentions. In support of this hypothesis, hierarchical regressions showed a negative relationship between desire for control and entrepreneurial intentions at T1 (β = −0.34, *p* < 0.05; see Table 2, Model 2); however, distrust of others was not significantly related to entrepreneurial intentions (β = −0.12, ns).

**FIGURE 1:**
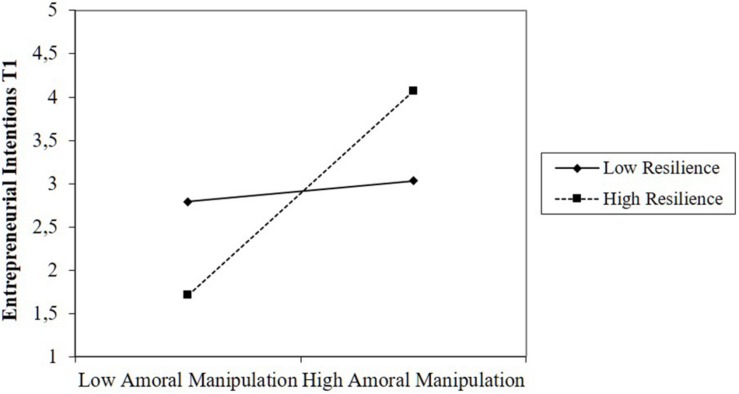
Moderating effect of resilience on the relationship between amoral manipulation and intentions.

**FIGURE 2:**
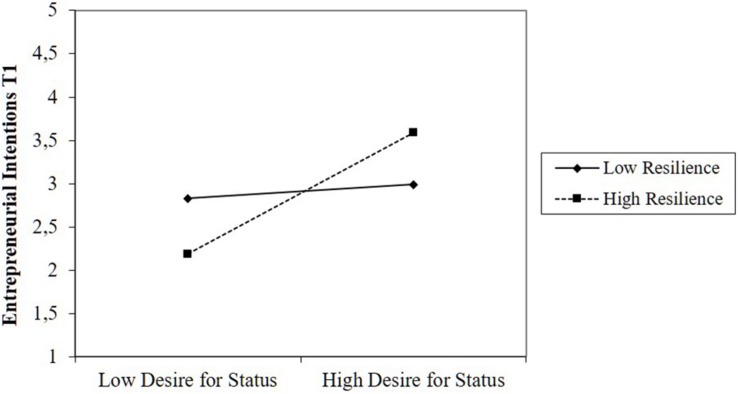
Moderating effect of resilience on the relationship between desire for status and intentions.

Hypotheses 3 and 4 predicted that team-level resilience would moderate the relationship between Machiavellianism and entrepreneurial intentions. We found partial support for the hypothesis that resilience increases the relationship between amoral manipulation and desire for status on the one hand and entrepreneurial intentions on the other hand (Hypothesis 3). The effect of amoral manipulation on intentions increases with increasing team resilience (β = 0.53, *p* < 0.01; Figure 1). However, the interaction term between desire for status and resilience on intentions was not significant, even though the plots displayed in Figure 2 indicated that the interaction is in the predicted direction: Desire for status was negatively related to intentions in teams with low levels of resilience, while this relationship was positive in teams with high levels of resilience.

**FIGURE 3:**
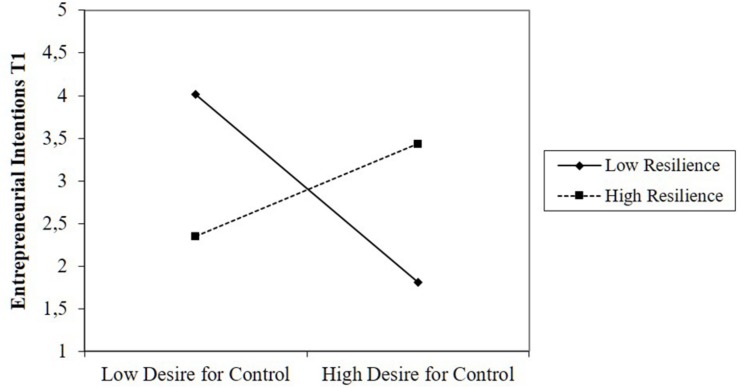
Moderating effect of resilience on the relationship between desire for control and intentions.

**FIGURE 4:**
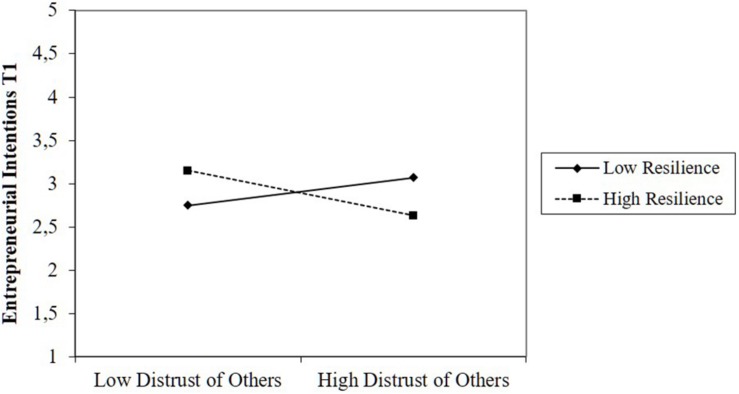
Moderating effect of resilience on the relationship between distrust of others and intentions.

**FIGURE 5:**
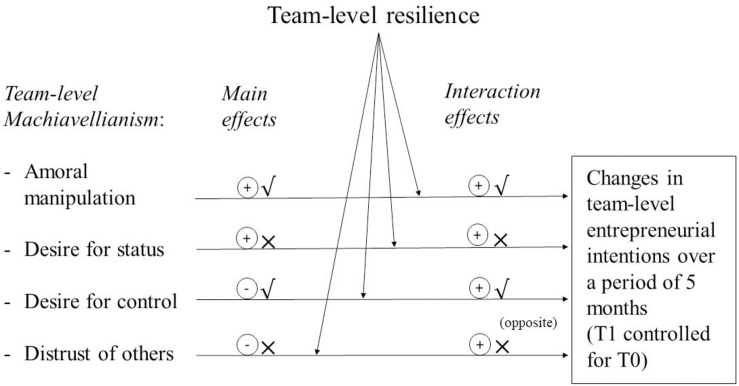
Summary of results and hypothesis testing.

As predicted in Hypothesis 4, we found that the effect of desire for control was moderated by resilience (β = 0.82, *p* < 0.01). When we plotted the interaction (Figure 3), we found that desire for control was negatively related to intentions in teams with low levels of resilience, while this relationship was positive in teams with high levels of resilience. However, while the interaction between distrust of others and resilience was significant, the plots indicated that the interaction was not in the expected direction (Figure 4). High distrust of others was positively related to intentions when resilience was low. Thus, we found partial support for Hypothesis 4. Figure 5 provides a summary of our results and the corresponding conclusions for our hypotheses.^1^

## Discussion

### Theoretical Implications

Academic interest in the psychology of entrepreneurship has developed in recent years, and some authors have suggested that entrepreneurs are not always heroic leaders with many positive characteristics but that there is also a negative side to entrepreneurship (Miller, 2015). This situation motivated us to explore whether the personality trait of Machiavellianism explains the emergence of entrepreneurial intentions in teams over time and how Machiavellianism affects those intentions at the team level. In addition, we investigated whether resilience helps teams with high Machiavellianism to develop start up intentions despite difficult team dynamics. We specifically chose to investigate resilience as it reflects a positive team characteristic that has received already some attention (see LePine et al., 2011) and might act as a resource for teams, helping them to buffer the potential negative effects of the dark trait of Machiavellianism. Our results support the notion that Machiavellianism (and its sub-dimensions) are related to entrepreneurial intentions when controlling for psychopathy and for narcissism. By statistical standards (Cohen, 1992), these effects were moderately large, the direct effects explaining 15% of the variance in entrepreneurial intentions and the interaction terms an additional 14% of the variance. Specifically, amoral manipulation and desire for status were positively related to intentions, whereas the relationship to intentions was negative for desire for control. In line with the findings of pioneering studies in this domain (Mathieu and St-Jean, 2013; Hmieleski and Lerner, 2016), these results indicate that entrepreneurial intentions are affected by the dark side of entrepreneurs’ personality – sometimes positively and sometimes negatively. Moreover, in teams these traits affect team level entrepreneurial intentions. Thus, entrepreneurship theory needs to take the dark characteristics of entrepreneurs in general and Machiavellianism in particular into account.

These results contribute to the psychological literature in entrepreneurship that has focused primarily on the positive characteristics of entrepreneurs so far (see a recent review by Kerr et al., 2018). However, more recent research indicates that such a romanticized view of entrepreneurship is misleading. For example, there are some indications that dark triad traits are related to entrepreneurial intentions, albeit sometimes based on unproductive motives (Hmieleski and Lerner, 2016). Some dimensions of Machiavellianism – such as amoral manipulation and desire for status – might be related to the need for power and the need for achievement (Jones and Paulhus, 2009), which are components of motivation theory (McClelland, 1961). Need for achievement and need for power have been found to be associated with positive aspects of work behavior, such as the motivation to lead, and with entrepreneurial activities (see Collins et al., 2004) but, at the same time, they are associated with negative and even unethical work behavior (Wolff and Keith, 2019). We similarly found differential effects for Machiavellianism and show that, despite having been linked to unethical or immoral action in previous research (O’Boyle et al., 2012), Machiavellianism (amoral manipulation and desire for control) can actually contribute to entrepreneurial intentions and behaviors under certain circumstances, for example, when individuals or teams are highly resilient.

Extending this stream of research, we showed that Machiavellianism is effective at the team level. Interestingly, team-level effect sizes were slightly higher than those of studies looking at the individual level. For example, Hmieleski and Lerner’s (2016) study reported between 7 and 16% explained variance when looking at individual-level dark triad traits and individual intentions, and Mathieu and St-Jean (2013), again looking at the individual level, found that narcissism explained 7% of the variance in intentions. Thus, the composition of individual traits in a team has an influence on the team and on team intentions. This is an important finding as most ventures are actually team efforts (Lazar et al., 2019), even though most trait studies in the domain of entrepreneurship examine individual agency.

While most of the empirical studies in the entrepreneurship team literature focus on the characteristics of teams (e.g., team homogeneity/heterogeneity) (for an overview, see Coad and Timmermans, 2014), only a few authors have discussed the disadvantages of entrepreneurial teams, such as the potential for inefficient communication, complex and endless decision processes, and conflicts between team members (Lechler, 2001; Ensley et al., 2002). In the organizational behavior literature, team dysfunctions such as group losses (Steiner, 1972), social loafing (Karau and Williams, 1993; Schippers, 2014), groupthink (Janis, 1972), and risk-shifting (Kogan and Wallach, 1967) are well documented, however, hardly any of those processes have been researched in the field of entrepreneurship. Ways of addressing dysfunctional entrepreneurial teams are to enhance the quality of social interaction within such teams (e.g., communication and cohesion), establish better work norms and mutual support, and provide more effective coordination and conflict resolution. All these processes might be affected by team-level Machiavellianism.

Our study also revealed that resilience was an important moderator. Significant two-way interactions between resilience and the sub-dimensions amoral manipulation, desire for control, and distrust of others showed that Machiavellianism was related differently to entrepreneurial intentions at the end of the course when resilience was high as compared to when it was low. These results indicate that the relationship between dimensions of Machiavellianism and outcomes is more complex than previously assumed. Therefore, our approach contributes to the personality approach to entrepreneurship, not only because we looked at the team level but also because we investigated the interactions between a dark personality trait and resilience. It is well established in personality theory that traits interact with other variables to predict outcomes (Mischel, 1968; Magnusson and Endler, 1977). However, such interactions have been almost ignored in entrepreneurship research (with one notable exception being; Hmieleski and Baron, 2009). While the interactions with two of the Machiavellianism sub-dimensions pointed in the expected direction (amoral manipulation and desire for control), the interaction between distrust of others and resilience was in the opposite direction; distrust of others had an adverse effect on entrepreneurial intentions when team resilience was high. In this sense, distrust of others seemed to act as a boundary condition for the positive buffering effects of resilience. Resilience could only act as a resource for teams and increase their entrepreneurial intentions when a minimum amount of trust of others was present in the team.

Also, we found in our study that men scored on average higher on Machiavellianism (except for the sub-dimension of desire for control), narcissism, and psychopathy as well as on entrepreneurial intentions than women. These results are in line with the extant literature on the dark traits (e.g., Christie and Geis, 1970; Paulhus and Williams, 2002) and on entrepreneurial intentions (e.g., Zhao et al., 2005; Wilson et al., 2007). While scholars on entrepreneurship point at gender differences on entrepreneurial self-efficacy (e.g., Wilson et al., 2007) and gender stereotypes (e.g., Gupta et al., 2009) as an explanation, our findings suggest that gender differences on dark personality traits might also play a role in explaining lower entrepreneurial intentions of women, compared to men.

Finally, our research also contributes to the debate whether dark triad traits form a unidimensional construct or whether there are some sub-dimensions of these traits that should be examined separately (Jakobwitz and Egan, 2006; Kam and Zhou, 2016). Specifically, there is evidence that various dark triad traits share variance and, therefore, there is a common underlying disposition of the dark triad (Volmer et al., 2019). However, when looking specifically at Machiavellianism, our results support the proposition that Machiavellianism needs to be conceptualized as a multidimensional construct consisting of amoral manipulation, desire for status, desire for control, and distrust of others (Dahling et al., 2009). Moreover, Machiavellians apply both hard and soft tactics as well as affiliative and confrontative manipulation tactics (Furnham et al., 2013), implying that there might be different processes underlying the overall construct. Our results reveal indeed differential relationships between the various sub-dimensions of Machiavellianism and intentions, thus suggesting that the sub-dimensions should be investigated separately in future research on entrepreneurship.

### Limitations and Future Directions

While a strength of the current study is that we tested the hypotheses with a large number of teams in a setting where entrepreneurial intentions were measured both at the beginning and end of the course, we recognize that only experimental studies can establish the causality implied in our research model. An obvious direction for future research would thus be to follow up this work with experimental designs (cf. LePine et al., 2011).

At the same time, it must be acknowledged that the teams in our study were student project teams, which is common practice when studying the dark side of entrepreneurship (e.g., Hmieleski and Lerner, 2016; Verheul et al., 2016) and also might be appropriate when looking at intentions (Hsu et al., 2017). However, future research should look at teams that are actually aiming to start up a business venture in order to establish whether our findings are generalizable to field contexts. While complementing our research with evidence from entrepreneurial teams would seem important, the limitations of our sample might result in a conservative assessment of the effect sizes, as our student teams are relatively homogeneous as compared to entrepreneurial teams and might thus decrease variance in our variables. Moreover, we relied on self-reports, but this should not be a major problem as our contribution lies to a large extent on lagged analyses and interactions. Those are not inflated by common source variance (Siemsen et al., 2010).

Also, we do not know exactly what happened in teams with high or low Machiavellianism and resilience. Importantly, although we alluded to several contagion-like processes within teams in the introduction section, research looking into the effects of personality on team processes is still at an early stage. For instance, team members that are high in distrust in others may influence each other, so that others also become distrustful of others. However, these processes have hardly been researched and are often mainly researched in terms of affect and group affective tone (i.e., the convergence of affect in a team; for a review see Collins et al., 2013). A similar line of research speaks of a ripple effect in teams and organizations (Barsade, 2002). For instance, the study of Barsade (2002) revealed that teams that experienced positive emotional contagion, also reported improved cooperation, decreased conflict, and increased perceived task performance. We thus need to know more about what core behavioral tendencies and processes in these teams facilitated the effects of Machiavellianism, e.g., by video-taping team interactions and coding team behaviors.

Further, future research should look at the long-term effects of dark traits such as Machiavellianism. Our study dealt with initial team formation and how the team functioned over time. Some new avenues for research might include the role of friendship within entrepreneurial teams and its association with performance and the partial break-up or dissolution of new venture teams (Francis and Sandberg, 2000). Also, future research could look into processes like coalition formation and competitive tactics in teams (Jehn et al., 2013). Since our venture projects were a mandatory activity as part of a course, only a few teams split up, but in real life, a substantial number of team-based ventures break up because of differences in objectives, commitment, and incentives (Wasserman, 2012). Therefore, we need to study entrepreneurial teams in a work context to investigate whether Machiavellianism would be functional or dysfunctional in the long term.

Next, another interesting avenue for future research would be to look at entrepreneurial teams in which only one (influential) team member has a high level of Machiavellianism and how such teams work with co-founders and employees (cf. Sy and Choi, 2013). For example, one study showed that team leaders high in Machiavellianism reduce trust and increase stress in employees (Belschak et al., 2018). However, entrepreneurial ventures need empowered employees to facilitate operational performance and innovation (Rauch, 2011). On the other hand, specifically resilient teams might be able to encourage firm-level learning and innovation, even though one team member being high in Machiavellianism. Future research could study how teams differing in terms of team composition with respect to Machiavellianism and resilience, develop over time, for instance in a field experiment, using methods that reveal real-time team interactions, such as diaries, interviews, and/or video-taping.

Finally, it is important to examine what it means if certain sub-dimensions of Machiavellianism (when controlling for narcissism and psychopathy), such as amoral manipulation and desire for control in- or decrease entrepreneurial intentions over time. Is this effect desirable? Are those teams who have higher intentions due to their high score on amoral manipulation better entrepreneurs, or do they provide a risk to other companies, future employees, and society? What does it mean if a team’s interest in entrepreneurship is based on a shared endorsement of manipulative tactics such as lying, cheating, sabotaging others, and other kind of unethical behavior (cf. Dahling et al., 2009)? Do we want these people to increase their motivation to become entrepreneurs? These are important questions that fall outside the scope of the current study and should be investigated in future research, and longitudinal research could come up with answers to these questions.

### Practical Implications

This study has (a) shed light on the relation between Machiavellianism on entrepreneurial intentions at the team level and (b) suggested how team resilience can have a positive role, providing a buffer against the negative effects of at least some of the dimensions of Machiavellianism. Given the extensive use of team projects in business education, our study has practical implications for entrepreneurship education and institutions that support entrepreneurship. There are numerous initiatives that aim to train and inspire people about entrepreneurship, and many of these rely on team projects (Rauch and Hulsink, 2015). The question remains as to whether such initiatives are stimulating the right people to become entrepreneurs. For instance, it is possible that entrepreneurship provides a unique setting that is attractive to people with a high level of amoral manipulation and a strong desire for status. Theoretically, Machiavellianism should become dysfunctional in the long run. The results of our study show that at least some dimensions of Machiavellianism are also dysfunctional in the short run. In particular desire for control seems to be a trait that not only stimulates undesirable and damaging work behaviors but is also detrimental to a team’s entrepreneurial intentions and needs to be buffered by high levels of team resilience. In this sense, companies would be well advised to choose the members of their entrepreneurial teams carefully. Moreover, the interaction with resilience also indicates that teams might develop entrepreneurial intentions despite a high level of Machiavellianism, which suggests that entrepreneurial teams should undertake resilience training (e.g., Seligman, 1990; Reivich and Shatté, 2002).

Although in our study we highlight the functional aspects of team-level Machiavellian dimensions, this does not mean we believe being high on those traits is necessarily desirable. The leadership literature suggests that those who obtain/assume positions of power do not necessarily benefit their organizations (Kaiser et al., 2008) or teams (Wolff and Keith, 2019). One should therefore be aware that teams high in amoral manipulation and/or desire for status might show increased entrepreneurial intentions, but this does not mean that these teams will necessarily show higher entrepreneurial effectiveness, and the long term consequences of Machiavellianism and resilience in teams are not well-known. So entrepreneurial teams should be monitored in terms of effectiveness, but also in terms of the means they use to achieve their ends in order to avoid undesirable (unethical) team behavior.

## Conclusion

Moving beyond the classical view of entrepreneurs where the emphasis has been on positive personality traits, psychological researchers have recently become interested in the dark side of entrepreneurship. Initial evidence from the study by Hmieleski and Lerner (2016) at the individual level showed that narcissism was positively related to entrepreneurial intentions, and that psychopathy and Machiavellianism were unrelated to students’ entrepreneurial intentions. Our findings contribute to this stream of research by going beyond the cross-sectional, individual-level approach and showing that, at the team level, various dimensions of Machiavellianism were related both positively (amoral manipulation and desire for status) and negatively (desire for control) to entrepreneurial intentions over time. Moreover, the interactional effects showed that team entrepreneurial intentions were predicted by a combination of team resilience and Machiavellianism, specifically the sub-dimensions amoral manipulation, desire for control, and distrust of others.

## Data Availability Statement

The datasets generated for this study are available on request to the corresponding author.

## Ethics Statement

This study was carried out in accordance with the recommendations of the Economics and Business Ethics Committee (University of Amsterdam) request number 20190506120556 with written informed consent from all subjects. All subjects gave written informed consent in accordance with the Declaration of Helsinki. The protocol was approved by the Economics and Business Ethics Committee (University of Amsterdam).

## Author Contributions

MS was involved in the research design, data gathering, data analyses, and contributed to the writing of all parts of the manuscript. AR contributed to the interpretation of the analysis, drafting, critical revision of the manuscript, and coordinating the writing. FB provided critical revisions of the manuscript. WH was involved in the actual research design and provided inputs to the “Theory and Hypotheses” and “Materials and Methods” sections. All authors agreed to the work and approved the final version of the manuscript.

## Conflict of Interest

The authors declare that the research was conducted in the absence of any commercial or financial relationships that could be construed as a potential conflict of interest.

## Footnotes

1 We ran the analyses also without controlling for psychopathy and narcissism. The results reveal that the explained variance is about the same as when including these controls (Δ*R*^2^ of direct effects is 0.14^∗∗^ and of interaction effects it is 0.11^∗∗^). The Beta weights are also all pointing in the same direction. However, the direct effect of amoral manipulation becomes insignificant as well as the interaction effect of resilience and amoral manipulation, when not controlling for psychopathy and narcissism. Significance levels of the other dimensions of Machiavellianism do not change. As it is considered good practice in research on Machiavellianism to include and control for narcissism and psychopathy in order to avoid ambiguous results (e.g., Paulhus and Williams, 2002; see Jones and Paulhus, 2009) we use and report results including these two control variables here.
